# Advances and challenges in immunotherapy for advanced esophageal squamous cell carcinoma

**DOI:** 10.3389/fimmu.2026.1739762

**Published:** 2026-04-10

**Authors:** Yishan Wang, Hui Li, Peiyan Zhao, Zhiming Li, Shuang Zhang

**Affiliations:** 1 The Affiliated Hospital of Jiangxi University of Chinese Medicine, Nanchang, China; 2 Medical Oncology Translational Research Lab, Jilin Cancer Hospital, Changchun, China; 3 Department of Thoracic Oncology, Jilin Cancer Hospital, Changchun, China

**Keywords:** biomarkers, drug resistance, esophageal squamous cell carcinoma, immune-related adverse events, immunotherapy

## Abstract

Esophageal squamous cell carcinoma (ESCC) is a highly prevalent and aggressive malignancy worldwide, associated with poor prognosis. Most patients are diagnosed at an advanced stage, where conventional chemotherapy offers limited therapeutic efficacy and is often accompanied by substantial toxicity. In recent years, immune checkpoint inhibitors (ICIs), particularly those targeting PD-(L)1 and CTLA-4, have emerged as cornerstone therapies in both first-line and subsequent treatment settings for advanced ESCC. Nevertheless, significant clinical challenges persist, including the complexity of mechanisms underlying immune resistance, suboptimal predictive performance of existing biomarkers, difficulties in the management of immune-related adverse events (irAEs), and underrepresentation of elderly patients in clinical trials. This review summarizes recent advances in immunotherapy for advanced ESCC, evaluating the clinical evidence supporting ICIs as monotherapy or in combination with agents such as anti-angiogenic drugs and tyrosine kinase inhibitors. It further discusses the therapeutic potential of novel approaches, including bispecific antibodies, CAR-T cell therapy, and next-generation ICIs, while addressing current treatment paradigms for elderly patients. The importance of comprehensive, longitudinal management of irAEs is emphasized. Additionally, this article provides an in-depth analysis of mechanisms contributing to immune resistance—such as loss of tumor neoantigens and dysregulation of key signaling pathways—and critically appraises the limitations of established biomarkers, including PD-L1 expression and tumor mutational burden (TMB), alongside emerging developments in biomarker discovery. In conclusion, while immunotherapy has significantly improved outcomes and expanded therapeutic prospects for patients with advanced ESCC, further research is required to elucidate resistance mechanisms, refine treatment strategies, and identify robust predictive biomarkers. These efforts are essential to advance precision medicine in ESCC and ultimately enhance long-term survival outcomes.

## 1 Introduction

According to GLOBOCAN 2022, esophageal cancer (EC) is the 11th most common cancer and the 7th leading cause of cancer deaths globally, representing 2.6% of new cancer cases and 4.6% of cancer-related deaths (1). EC is primarily classified into two subtypes: esophageal squamous cell carcinoma (ESCC, 85%) and esophageal adenocarcinoma (EAC), with distinct regional characteristics (2). ESCC is particularly prevalent in Eastern Europe and Asia, and its occurrence and development are significantly associated with risk factors such as tobacco exposure and alcohol intake (3, 4). Due to the insidious early symptoms of this disease, approximately 70% of patients are diagnosed at an advanced stage, missing the opportunity for radical surgical treatment (5).

From 1940 to 1990, the treatment of advanced EC mainly relied on fluorouracil- and platinum-based chemotherapy, which yielded a median overall survival (mOS) of only 7 to 13 months and a 5-year survival rate below 10% (1, 6). Although HER2-targeted therapies have significantly improved the treatment of EAC, especially gastroesophageal junction cancer, since the early 2000s, the treatment of advanced ESCC has long been stuck in the era of traditional chemotherapy. Not only is the objective response rate (ORR) limited [about 30-40% (first-line)], but it is also accompanied by significant toxic side effects such as bone marrow suppression and gastrointestinal toxicity. There is an urgent need for breakthrough treatment strategies in clinical practice (7).

Immunotherapy, particularly immune checkpoint inhibitors (ICIs), has revolutionized cancer treatment. In 2011, ipilimumab, the first ICI targeting CTLA-4, was approved by the U.S. Food and Drug Administration (FDA) for unresectable or metastatic melanoma, marking the beginning of a new era in cancer immunotherapy. Subsequently, ICIs have also achieved significant progress in the field of ESCC. Following the positive results of key phase III trials such as KEYNOTE-181 and ATTRACTION-3 in the second-line setting, the research focus has gradually shifted to first-line treatment. Landmark studies including CheckMate 648 and KEYNOTE-590 have further established the standard role of immunotherapy combined with chemotherapy in the first-line treatment of advanced ESCC.

Although ICIs have made breakthrough progress in treating ESCC, significant challenges remain. The predictive value of current biomarkers (such as PD-L1, TMB, and MSI) is limited, and a reliable predictive system for therapeutic efficacy is lacking. Primary or acquired resistance prevents most patients from benefiting from immunotherapy. The elderly population is underrepresented in clinical studies, and the efficacy and safety of immunotherapy in this subgroup warrant further investigation. In addition, the management of immune-related adverse events (irAEs) lacks standardized guidelines.

Against this background, this review focuses on the latest advances in immunotherapy for advanced ESCC, including combination therapeutic strategies, the development of novel immunotherapeutic approaches, the current status and challenges of immunotherapy in elderly patients, and the monitoring and management of irAEs. Furthermore, we discuss the key challenges in achieving precision immunotherapy, including the identification of effective biomarkers and the exploration of resistance mechanisms.

## 2 Application of immunotherapy in advanced ESCC

### 2.1 Second-line and later-line immunotherapy for advanced ESCC

The selection of second-line and later-line treatment strategies for advanced ESCC should be guided by the patient’s prior immunotherapy history. In patients who have not previously received ICIs, clinical evidence demonstrates that ICIs monotherapy offers significant advantages over traditional chemotherapy. KEYNOTE-028 was a global, multicenter, multi-cohort Phase Ib study designed to evaluate the efficacy and safety of pembrolizumab in patients with previously treated, PD-L1-positive advanced EC. The study enrolled a total of 23 patients, and the results showed an ORR of 30%, with a median duration of response (DOR) of 15 months and a median progression-free survival (PFS) of 1.8 months (8). The subsequent KEYNOTE-180 Phase II study further assessed the efficacy and safety of pembrolizumab in patients with advanced EC who had progressed after two or more lines of prior therapy. The primary endpoint of the study was ORR. Results indicated an ORR of 9.9% in the overall population, with an ORR of 14.3% in the ESCC subgroup. The median overall survival (OS) for the entire cohort was 5.8 months, and the median PFS(mPFS) was 2.0 months (9).

Another pivotal study, ATTRACTION-1, was a multicenter, single-arm, Phase II clinical trial that evaluated the efficacy and safety of nivolumab in patients with advanced ESCC who had previously received fluoropyrimidine, platinum, or taxane-based chemotherapy, or who were intolerant to chemotherapy. The majority of enrolled patients had received three or more prior lines of therapy. Results showed that among evaluable patients, the ORR was 17%, the disease control rate was 42%, and the median PFS was 1.5 months (95% CI: 1.4-2.8 months). The incidence of grade ≥3 TRAEs was 17% (10). These studies provide important evidence-based medical support for PD-1 inhibitors as a viable treatment option for later-line therapy in advanced ESCC, while also confirming their significant potential to improve patient prognosis in this clinically challenging treatment-refractory population.

Building on the positive outcomes of the KEYNOTE-180 trial, the subsequent phase III KEYNOTE-181 study provided the first evidence demonstrating a survival benefit associated with immune monotherapy in the second-line treatment setting. Among patients with advanced ESCC and PD-L1 combined positive score (CPS) ≥10, pembrolizumab significantly improved mOS compared to chemotherapy (9.3 vs. 6.7 months). Subgroup analyses revealed a more pronounced survival benefit in Asian patients, particularly those from China, with a 2.8-month prolongation in mOS for CPS ≥ 10 ESCC patients compared to chemotherapy. These findings led to FDA approving pembrolizumab for second-line treatment of CPS ≥ 10 ESCC in 2019 (11). Further phase III trials, including ATTRACTION-3, ESCORT, and RATIONAL-302, consistently confirmed the survival advantage of ICIs (mOS: 8.3-10.9 months vs 6.2-8.4 months). Among these Phase III trials, KEYNOTE-181 enrolled approximately 38.5% Asian patients, with squamous cell carcinoma accounting for 64% of the cohort. ATTRACTION-3 and RATIONALE-302 were studies predominantly involving Asian populations, enrolling only patients with squamous cell carcinoma, while ESCORT was a study specifically targeting Chinese ESCC patients. Although the primary endpoint for all these studies was OS, the KEYNOTE-181 study specifically assessed OS in esophageal squamous cell carcinoma patients with CPS ≥ 10. Consequently, pembrolizumab is indicated only for patients with PD-L1 CPS ≥ 10 in the second-line and later-line settings, whereas the application of nivolumab in ESCC is not restricted by PD-L1 expression levels (12–14).

In terms of safety, the incidence of grade ≥3 TRAEs in the ICIs group (16%–19.8%) was significantly lower than that in the chemotherapy group (23%–55.8%). However, isolated cases of fatal immune-related adverse events (e.g., myocarditis, interstitial pneumonia) were still sporadically reported across studies, underscoring the need for enhanced comprehensive monitoring and individualized management of irAEs in clinical practice.

Following the failure of first-line immunochemotherapy, developing effective subsequent treatment strategies has emerged as an increasingly prominent and unmet clinical need in current ESCC practice. Currently, clinical guidelines recommend second-line and later-line systemic therapy options including docetaxel, paclitaxel, or irinotecan (15). Some small-scale retrospective studies suggest that nanoparticle albumin-bound paclitaxel monotherapy can achieve an ORR of up to 33.3% (16). Irinotecan combined with 5-fluorouracil has also demonstrated certain activity (17). However, these data are supported by low-level evidence, generally limited by small sample sizes and inadequate control of confounding factors. More critically, most patients have developed acquired resistance following first-line immunotherapy, and whether subsequent chemotherapy sensitivity is altered by prior immune exposure remains mechanistically unclear. Against this backdrop, exploring novel combination strategies (such as anti-angiogenic agents and ADC drugs) and developing innovative targeted therapies have become core directions for second-line and later-line research.

#### 2.1.1 ICIs-based combination therapy

In the field of second-line and later-line immunotherapy combinations, current research primarily focuses on ICIs combined with anti-angiogenic TKIs or anti-EGFR monoclonal antibodies. However, the vast majority are exploratory Phase I/II single-arm studies, with no confirmatory Phase III data released to date. Existing Ib/II phase studies report ORR ranging from 10.2% to 32.6% and mPFS from 1.5 to 9.4 months, demonstrating significant heterogeneity in efficacy across different trials. This variability is not only related to differences in the mechanisms of the combination drugs but is also confounded by the heterogeneity of prior immunotherapy exposure status among enrolled populations. Some studies have enrolled “pre-treated” patients without clearly distinguishing between “prior ICIs exposure” and “ICIs-naïve” groups—two populations with fundamentally different biological behaviors (18–23). Of note, a Phase II study reported at the 2025 ASCO Annual Meeting demonstrated that the second-line regimen of nimotuzumab combined with camrelizumab achieved an ORR of 32.6% and an mOS of 12.68 months, with a trend toward greater benefit observed in the PD-L1 CPS < 1 population. However, this study had a single-arm design, a small sample size of only 38 patients, and was presented in abstract form without formal peer-reviewed publication. Therefore, it cannot currently serve as evidence-based support for the routine clinical application of this regimen (22).

#### 2.1.2 Bispecific antibody

Monotherapy directed at a single target often encounters substantial challenges, as tumor cells can adapt to and evade the therapeutic effects of ICIs through diverse molecular and cellular mechanisms (24). Combined therapy can make up for the insufficiency of single target therapy, exert a synergistic anti-tumor effect, overcome tumor heterogeneity, and reduce the risk of drug resistance. However, this also means that combination therapy may lead to the accumulation of side effects, among which drug interactions or additive effects may increase the incidence of side effects. Bispecific antibodies (BsAbs), engineered via cell fusion or recombinant DNA technology, are capable of simultaneously binding to two distinct antigens or separate epitopes of the same antigen, thereby improving therapeutic efficacy and minimizing the risk of potential adverse effects (25). Cadonilimab (PD-1/CTLA-4) is the world’s first tumor immunotherapy bispecific antibody (BsAb) approved for marketing. It has currently received approval from China’s National Medical Products Administration (NMPA) in the fields of cervical cancer and gastric cancer, and has been included in the CSCO guidelines recommendations (26). In the field of ESCC, this drug is currently undergoing critical clinical evaluation: the ESCC cohort of the COMPASSION-03 study showed an ORR of 18.2% and ≥ grade 3 TRAE rate of 28%. Numerically, this efficacy did not significantly surpass historical data from second-line PD-1 monotherapy (**Table 1**), and the toxicity appeared additive rather than synergistically reduced. This phenomenon suggests that the theoretical advantage of BsAbs in achieving “reduced toxicity and enhanced efficacy” in solid tumors requires further in-depth exploration in ESCC (27). Currently, two phase II clinical studies are underway to investigate the value of cadonilimab combined with radiotherapy or anti-angiogenic TKIs in the immunotherapy-resistant ESCC population (NCT06681285, NCT05732662). Furthermore, novel BsAbs targeting PD-1/LAG-3 and PD-1/TIM-3 have also entered early clinical stages (NCT04140500, NCT04785820). The results of these studies will directly address a core scientific question: Are BsAbs a formulation improvement over existing combination regimens (PD-1 + CTLA-4), or do they represent a genuine mechanistic innovation?

**Table 1:** Key clinical trials evaluating second-line and later-line therapies for ESCC.

Study(year)	Study design	Biomarker selection	Primary endpoint	PFS(mo)	OS(mo)	ORR	Grade≥3 AEs	N	Regioin	Elderly subgroup (≥65y)
Phase III confirmatory trial										
KEYNOTE-181 (11)(2020)	Pembrolizumab	PD-L1 CPS	OS	2.0	5.8	14.3%	12.4%	121	Global	Accounting for 44.3% OS HR:≥65y 0.76 (0.55-1.06); <65y 0.81 (0.61-1.07)
ATTRACTION-3 (12)(2019)	Nivolumab vs Chemo	PD-L1	OS	1.7 vs 3.4	10.9 vs 8.4	19% 22%	16% vs 23	419	Global	Accounting for 47% OS HR:≥65y 0.86 (0.63-1.16); <65y 0.65 (0.47-0.89)
ESCORT (13) (2020)	Camerizumab vs chemo	PD-L1 TPS	OS	1.9 vs 1.9	8.3 vs 6.2	7.4 mo 3.4 mo	19% vs 40%	457	China	Accounting for 46% OS HR:≥65y 0.60 (0.39-0.91); <65y 0.75 (0.59-0.96)
RATIONAL-302 (14) (2022)	Tislelizumab vs chemo	TAP	OS	1.6 vs 2.1	8.6 vs 6.3	20.3% 9.8%	18.8% 55.8%	512	Global	Accounting for 38.7% OS HR:≥65y 0.64 (0.47-0.89); <65y 0.73 (0.57-0.93)
Exploratory trials (Phase I/I b/II)										
ATTRACTION-1 Phase II (10) (2017)	Nivolumab	–	ORR	2.9	10.8 mo	22%	26%	65	Japan	–
KEYNOTE-028 Phase Ib (8) (2018)	Pembrolizumab	PD-L1	ORR and safety	1.8	7.0 mo	30%	17%	23	Global	–
KEYNOTE-180 Phase II (9) (2019)	Pembrolizumab	PD-L1 CPS	ORR	2.0	5.8 mo	14.3%	12.4%	121	Global	–
CAP 02 Phase II (19) (2024)	Apatinib +Camrelizumab	PD-L1 CPS	ORR	4.6	7.5 mo	10.2%	34.7%	49	China	–
Ning et al Phase Ib (20)(2024)	benmelstobart +AL2846	–	ORR and safety	3.22	5.98 mo	11.1%	30.3%	33	China	–
Liu et al Phase II (21) (2024)	HLX07	EGFR H-Score	PFS and ORR	1.5	5.8 mo	15%	10%	20	China	–
Xia et al. Phase II (22) (2025)	Camrelizumab + nimotuzumab	–	ORR	9.4	12.68 mo	32.6%	9%	38	China	–
Muro et al Phase Ib (23) (2022)	futibatinib + pembrolizumab	–	ORR and DLT	–	–	20%	–	30	Japan	–
COMPASSION-03 Phase Ib/II (27) (2023)	cadonilimab	PD-L1 CPS	ORR	Ib: 1.6 II: 3.5	Ib: 15.2 mo II: 9.4 mo	Ib: 7% II: 18.2%	28%	Ib: 83 II: 157	China	–
Gutierrez et al Phase I (28) (2025)	INCAGN02390 (TIM-3 inhibitor)	–	MTD	1.9	–	2.5%	8%	40	America	–
RAMONA Phase II (29)(2022)	Nivolumab± ipilimumab	PD-L1 CPS,TPS	OS	2.7	7.2 m	18.2%	20%	66	Germany	–

ESCC, esophageal squamous cell carcinoma; mo, months; CPS, combined positive score; TPS, tumor proportion score; TAP, tumor area positivity; nab-paclitaxel, nanoparticle albumin bound-paclitaxel;5-Fu:5-fluorouracil.

#### 2.1.3 New immune checkpoint inhibitors

Following PD-(L)1 inhibitor treatment, the compensatory upregulation of alternative immune checkpoints such as LAG-3, TIM-3, and T cell immunoreceptor with Ig and ITIM domains inhibitor (TIGIT) is considered a key mechanism mediating acquired resistance to immunotherapy. Theoretically, dual immune checkpoint blockade strategies could effectively mitigate T-cell exhaustion and tumor progression, thereby inducing durable antitumor immune responses. However, early clinical data for agents targeting these novel checkpoints in ESCC remain discouraging. A phase I study of a TIM-3 inhibitor demonstrated limited single-agent antitumor activity (ORR of only 2.5%) in heavily pretreated patients with advanced solid tumors, including ESCC, and failed to show immunopharmacodynamic evidence of induced T-cell proliferation (28). Combining TIM-3 inhibitors with PD-(L)1 inhibitors may enhance their antitumor efficacy (30). The AdvanTIG-203 study, which enrolled ESCC patients with PD-L1 TAP ≥ 10%, aimed to evaluate the efficacy and safety of ociperlimab combined with tislelizumab. Results showed that compared to tislelizumab monotherapy combined with placebo, the combination regimen demonstrated a trend toward improved ORR, the primary endpoint (30.6% vs. 20.6%; HR: 0.93), but did not significantly improve PFS (31).

#### 2.1.4 Chimeric antigen receptor T-cell immunotherapy

Chimeric antigen receptor T-cell (CAR-T) therapy represents a groundbreaking immunotherapeutic strategy based on genetic engineering, enabling precise and targeted eradication of malignant cells through the expression of chimeric antigen receptors that specifically recognize tumor-associated antigens.

In the ESCC field, current clinical research remains concentrated in the Phase I/II dose-finding stage, targeting antigens including MUC1, CEA, EpCAM, and NY-ESO-1 (NCT03941626, NCT03706326, NCT06010862, NCT03013712). Although CAR-T therapy represents a significant advancement in the field of immunotherapy, its application in solid tumor treatment still faces numerous challenges, with efficacy significantly constrained by multiple factors, including tumor antigen heterogeneity, insufficient infiltration capacity of CAR-T cells into tumor tissue, and the immunosuppressive tumor microenvironment (TME) (32). These factors collectively limit the antitumor efficacy of CAR-T therapy in solid tumors and remain major obstacles to its clinical translation. Additionally, the complex manufacturing process and high production costs also restrict the widespread application of this technology. Beyond technical limitations, treatment-related toxicities represent another important factor affecting its clinical translation, potentially further constraining patient clinical management and treatment accessibility. The most common and clinically significant adverse events include cytokine release syndrome and immune effector cell-associated neurotoxicity syndrome.

### 2.2 Advances in first-line immunotherapy for advanced ESCC

Chemotherapy, as the first-line standard treatment for advanced EC, has seen extremely limited progress over the past few decades. The emergence of immunotherapy has brought groundbreaking changes to the first-line treatment of EC. The KEYNOTE-590 study was the first phase III randomized controlled trial to demonstrate that first-line immunotherapy plus chemotherapy was significantly superior to chemotherapy alone for advanced EC, establishing it as the new standard of care for unresectable metastatic EC (33). Subsequently, multiple phase III clinical studies investigating PD-1/PD-L1 inhibitors combined with chemotherapy as first-line treatment for ESCC have successively reported positive results, further consolidating the position of chemo-immunotherapy in the first-line setting. With extended follow-up, long-term survival data from KEYNOTE-590 and CheckMate 648 show that the 5-year OS rate in the chemo-immunotherapy groups reaches 12%–13.8%, significantly higher than the 3.7%–9% observed in the chemotherapy groups, further validating its efficacy and safety (33, 34).

Although current PD-1/PD-L1 inhibitors combined with chemotherapy have yielded positive results in the first-line treatment of ESCC, these studies exhibit significant heterogeneity in multiple aspects, including study design, population selection, chemotherapy regimens, and the choice of efficacy-related biomarkers. For instance, KEYNOTE-590 was a global study with approximately equal proportions of Asian and non-Asian participants, of which 73% had squamous cell carcinoma. CheckMate 648 (34)and RATIONALE-306 (7) were also global studies, with Asian patients accounting for approximately 70%, rendering these studies applicable to a broader population. In contrast, GEMSTONE-304, JUPITER-06, ASTRUM-007, and ESCORT-1st were studies specifically targeting Chinese patients with ESCC (35–38). Meanwhile, although the ORIENT-15 (39) study was designed as a global trial, the vast majority (98%) of its enrolled patients were Chinese with ESCC, suggesting that the regimen from this study may be more specifically applicable to the Chinese ESCC population.

Different chemotherapeutic agents exert distinct effects on the tumor immune microenvironment. Cisplatin combined with 5-FU and cisplatin combined with paclitaxel are both available first-line chemotherapy regimens for esophageal cancer. Preclinical studies suggest that 5-FU and paclitaxel have different regulatory effects on immune cells within the tumor microenvironment, indicating that selecting the “optimal chemotherapy partner” with synergistic effects when combined with PD-1/PD-L1 inhibitors may be crucial for enhancing antitumor efficacy.

Currently, multiple clinical studies investigating first-line immunotherapy combined with chemotherapy for EC have adopted different chemotherapy regimens. For example, the ORIENT-15 study allowed investigators to choose between DDP plus paclitaxel or DDP plus 5-FU; however, only approximately 6% of enrolled patients actually received the latter regimen. In the GEMSTONE-304 and ESCORT-1st studies, although different chemotherapy backbones were employed, the hazard ratios (HR) for death in the immunotherapy-plus-chemotherapy groups versus the control groups were similar. At present, head-to-head comparisons regarding the optimal chemotherapy backbone in immunotherapy combinations are still lacking. Therefore, further preclinical and clinical investigations are warranted to determine the most effective chemotherapy combination strategy in first-line immunotherapy for EC.

Furthermore, these phase III studies investigated the predictive value of PD-L1 expression for the efficacy of first-line immunotherapy. However, substantial heterogeneity in the PD-L1 assays used—including differences in testing platforms, antibody clones, definitions of positivity, and cutoff values—precludes direct comparison of their findings. Overall, most studies demonstrated a correlation between PD-L1 expression and the efficacy of immunotherapy, with exceptions such as the ORIENT-15 study, which suggested that the efficacy of sintilimab plus chemotherapy was not significantly associated with PD-L1 expression levels.

The KEYNOTE-590 study, although enrolling an unselected population, included OS in patients with squamous cell carcinoma and a CPS ≥ 10 as one of its primary endpoints and achieved positive results. In contrast, the ASTRUM-007 study was conducted in a selected population with PD-L1 CPS ≥ 1 and also observed significant benefits in both OS and PFS. A meta-analysis incorporating 10 phase III randomized controlled trials (including five in the first-line setting) demonstrated that immune checkpoint inhibitors provide a consistent benefit in reducing the risk of death for patients with ESCC, and this benefit was correlated with PD-L1 CPS status. However, further exploration of other immunotherapy biomarkers is still needed in the patient subgroup with CPS < 10 (40). Another meta-analysis, which included 17 phase III randomized controlled trials (including nine in the first-line setting), evaluated the correlation between PD-L1 expression status and treatment efficacy. It found that in ESCC, tumor proportion score (TPS) was the strongest predictive factor for efficacy, whereas in esophageal adenocarcinoma (EAC), CPS was the strongest predictor, second only to MSI-H status (41). Overall, whether ESCC patients with low PD-L1 expression levels can truly derive benefit from PD-1/PD-L1 inhibitors combined with chemotherapy remains controversial and requires further clarification through additional studies.

Although PD-1/PD-L1 inhibitors combined with chemotherapy have significantly improved the survival prognosis of patients with advanced esophageal cancer, the efficacy still needs further enhancement, prompting researchers to conduct numerous investigations. One approach involves adding new agents to the current immunochemotherapy backbone to achieve synergistic effects. TIGIT is a co-inhibitory receptor that suppresses T cell proliferation and cytotoxic function. Concurrent blockade of the TIGIT and PD-1/PD-L1 pathways exerts synergistic antitumor effects. The SKYSCRAPER-08 study evaluated the efficacy and safety of combining the TIGIT inhibitor tiragolumab and atezolizumab with chemotherapy for the first-line treatment of esophageal squamous cell carcinoma. Results showed that this regimen significantly improved patients’ PFS and OS, offering a novel immunotherapy strategy for ESCC (42). However, it is noteworthy that the control arm in this study was chemotherapy alone, rather than the current standard of immunochemotherapy. Therefore, whether this combination model is truly superior to the existing standard regimen remains to be validated.

In addition, the combination of bispecific antibodies and chemotherapy has also been preliminarily explored in ESCC. In a phase II study of cadonilimab, a bispecific antibody targeting both PD-1 and CTLA-4, combined with chemotherapy for the first-line treatment of ESCC, the ORR reached 81.4%, with a median PFS of 7.10 months. Importantly, the efficacy was not limited by PD-L1 expression levels; among patients with PD-L1 CPS < 1, the ORR was still 87.5%, and the median PFS was 8.41 months, suggesting its potential as a treatment option for patients with low PD-L1 expression (43). SHR-1701 is a bifunctional fusion protein targeting PD-L1 and TGF-βRII. In a single-arm phase II study of SHR-1701 combined with cisplatin and nab-paclitaxel for the first-line treatment of advanced ESCC, the ORR was 62.5%, the median PFS was 10.8 months, and the median OS was 26.1 months. The incidence of grade ≥3 treatment-related adverse events was 45.8%, demonstrating significant antitumor activity and durable survival benefits (44). Concurrently, the combination of PD-1/PD-L1 inhibitors with anti-angiogenic agents has also become a direction of exploration. The LEAP-014 study evaluated the efficacy and safety of lenvatinib combined with pembrolizumab and chemotherapy. However, compared to the standard treatment (pembrolizumab plus chemotherapy), this combination strategy failed to improve OS and did not meet its primary endpoint, revealing its limitations in the treatment of esophageal cancer (45). Approximately 50-70% of ESCC patients exhibit high EGFR expression, which is associated with poor prognosis. Therefore, combining anti-EGFR monoclonal antibodies with PD-1/PD-L1 inhibitors and chemotherapy also represents a promising strategy. In the realm of local treatment combinations, adding local radiotherapy to existing systemic therapy has also shown potential. The ESO-Shanghai13 study found that compared to systemic therapy alone, the addition of local radiotherapy significantly prolonged median PFS (15.3 vs. 6.4 months, HR = 0.26), reducing the risk of disease progression by 74%; although the median OS in the combination therapy group was not reached, it reduced the risk of death by 58% (HR: 0.42, NR vs. 18.6 months) (46).

On the other hand, exploratory efforts in chemotherapy-free regimens have also achieved preliminary progress, particularly in dual immune checkpoint inhibitor combinations and strategies combining immunotherapy with targeted therapy. The CheckMate 648 study demonstrated that nivolumab combined with the CTLA-4 inhibitor ipilimumab significantly improved OS compared to chemotherapy (34). In the realm of immunotherapy plus targeted therapy, the phase II ALTER-E003 study evaluated the efficacy of anlotinib combined with the PD-L1 inhibitor bemelsuobai (TQB2450) as a first-line treatment for advanced ESCC. The results showed a median PFS of 15.74 months, a median OS of 20.57 months, and an ORR of 56.5%, offering a novel approach for chemotherapy-free strategies (47).

In summary, research on first-line and subsequent-line immunotherapy for advanced ESCC has evolved into a diversified therapeutic landscape (see **Tables 1**–**3**). Nevertheless, alongside the assessment of clinical efficacy, the management of irAEs remains a critical consideration in clinical practice. Current evidence indicates that the incidence of irAEs exhibits substantial variability (ranging from 10% to 90%), among which the incidence of grade 3 or higher severe adverse events falls within the range of 2.5% to 18% (48). Generally, irAEs tend to occur within several weeks to months following the initial administration of ICIs, exhibiting a delayed onset characteristic. Notably, these toxic reactions may continue to occur even after the completion or discontinuation of treatment. The underlying mechanisms are complex and not yet fully elucidated; but it is known to be related to abnormal T-cell activation attacking normal tissues and changes in peripheral blood B-cell subsets (49, 50). Due to the disruption of the body’s immune balance by ICIs, these adverse reactions can affect multiple organ systems, encompassing nearly all organs including but not limited to the skin, endocrine system, gastrointestinal tract, nervous system, and hematopoietic system (51–53). In some cases, especially among patients with pre-existing conditions, these events may become life-threatening (54).

**Table 2:** Key clinical studies on first-line treatment for ESCC.

Study(year)	Study design	Biomarker selection	Primary endpoint	PFS(mo)	OS(mo)	ORR	Grade≥3 AEs	N	Regioin	Elderly subgroup (≥65y)
Phase III confirmatory trial										
RATIONALE-306 (7)(2023)	Tislelizumab vs Placebo+CT	PD-L1 TAP PD-L1 CPS	OS	7.3 vs 5.6	17.2 vs 10.6	63% 42%	67% vs 64%	649	Global	Accounting for 48% OS HR:≥65y 0.62 (0.47-0.82); <65y 0.73 (0.56-0.95)
KEYNOTE-590 (33)(2021)	Pembrolizumab vs Placebo+ CT	PD-L1 CPS	PFS OS	6.3 vs 5.8	12.6 vs 9.8	–	72% vs 68%	749	Global	Accounting for 43% OS HR:≥65y 0.69 (0.53-0.89); <65y 0.76 (0.61-0.95)
Checkmate-648 (34)(2022)	Nivolumab+ CT vs Nivolumab+ Ipilimumab vs CT	PD-L1 PD-L1 CPS	PFS OS	6.9 vs 4.0 vs 4.4	15.4 vs 13.7 vs 9.1	53% 35% 20%	47% vs 32% vs 36%	970	Global	Accounting for 48% OS HR: Nivolumab+CT ≥65y 0.40(0.27-0.61), <65y 0.68(0.47-0.97);Nivolumab+Ipilimumab ≥65y 0.44(0.29-0.68) <65y 0.81(0.57-1.14)
GEMSTONE-304 (35)(2024)	Sugamlimab+CT vs Placebo+CT	PD-L1	PFS OS	6.2 vs 5.4	15.3 vs 11.5	60.1% 45.2%	51.3% vs 48.4%	540	China	Accounting for 39.7% OS HR:≥65y 0.77 (0.52-1.15); <65y 0.67 (0.50-0.92)
JUPITER-06 (36)(2022)	Toripalimab+CT vs Placebo+CT	PD-L1 CPS	PFS OS	5.7 vs 5.5	17 vs 11	69.3% 52.1%	73.2 vs 70%	514	China	Accounting for 38% OS HR:≥65y 0.62 (0.36-1.07); <65y 0.59 (0.41-0.86)
ASTRUM-007 (37)(2023)	Serpluliumab vs Placebo+CT	PD-L1 CPS	PFS OS	5.8 vs 5.3	15.3 vs 11.8	57.6% 42.1%	53% vs 48%	551	China	Accounting for 46% OS HR:≥65y 0.76 (0.52-1.12); <65y 0.62 (0.45-0.87)
ESCORT-1 (38) (2021)	Camerizumab vs Placebo+CT	PD-L1 TPS	PFS OS	15.3 vs 12	6.9 vs 5.6	72.1% 62.1%	63.4% vs 67.7%	490	China	Accounting for 32.6% OS HR:≥65y 0.65 (0.44-0.95); <65y 0.73 (0.55-0.96)
ORIENT-15 (39) (2022)	Sintilimab vs Placebo+CT	PD-L1 CPS PD-L1 TPS	PD-L1 CPS≥10 OS	8.3 vs 6.4 (PD-L1 CPS≥10)	17.2 vs 13.6 (PD-L1 CPS≥10)	66% 45%	60% vs 55%	659	Global	Accounting for 41% OS HR:≥65y 0.54 (0.38-0.77); <65y 0.70 (0.54-0.92)
SKYSCRAPER-08 (42) (2024)	Atezo + Tira + CT vs Placebo + CT	–	PFS OS	6.2 vs 5.4	15.7 vs 11.1	59.7% 45.5%	62.2% vs 57.3%	461	Asia	–
Exploratory trials (Phase II)										
Liu et al (21) (2024)	Serplulimab + HLX07+CT	EGFR H-Score	PFS OS	7.8 vs 1.5	Not reach	60%	43.4%	30	China	**-**
ESO-Shanghai 13 (46) (2024)	Systemic + RT vs Systemic	–	PFS	15.3 vs 6.4	Not reach vs 18.6	–	47% vs 41%	104	China	**-**
ALTER-E-003 (47) (2025)	Anlotinib + benmelstobart	NGS, PD-L1 CPS PD-L1 TPS	ORR	15.74	20.57	56.5%	28.3%	46	China	**-**

ESCC, esophageal squamous cell carcinoma; CPS, combined positive score; TPS, tumor proportion score; TAP, tumor area positivity; Atezo, atezolizumab; Tira: tiragolumab; CT: chemotherapy; RT: radiotherapy; mo: months; Systemic therapy: chemotherapy or Immunotherapy with or without chemotherapy.

**Table 3:** Ongoing clinical trials.

Treatment regimen	Cancer type	Clinical phase	Status	Clinical trials.gov identifier
Camrelizumab + Apatinib vs Camrelizumab	ESCC	Phase III	Unknown status	NCT05049681
BL-B01D1(ADC)	ESCC	Phase III	recruiting	NCT06304974
KC1036(TKI) vs CT	EC	Phase III	recruiting	NCT06194734
Cadonilimab+ Anlotinib+RT	ESCC	Phase II	not recruiting	NCT06681285
AK104(BsAbs) vs AK104+SBRT	ESCC	Phase I/II	not recruiting	NCT05732662
RO7247669(BsAbs)	Solid tumor	Phase I	Active, not recruiting	NCT04140500
Lomvastomig vs tobemstomig vs nivolumab	ESCC	Phase II	Completed	NCT04785820
Tislelizumab + Ociperlimab vs Tislelizumab	ESCC	Phase II	Completed	NCT04732494
CAR-T/TCR-T cells immunotherapy	Solid Tumors	Phase I/II	Unknown status	NCT03941626
Anti-MUC1 CAR-T cells vs CAR-T + PD-1 Knockout T cells vs PD-1 knockout Engineered T cells	EC	Phase I/II	Unknown status	NCT03706326
CEA CAR-T cells	Solid Tumors	Phase I	recruiting	NCT06010862
EpCAM CAR-T cell	Solid Tumors	Phase I/II	Unknown status	NCT03013712
Pembrolizumab+Lenvatinib+CT	EC	Phase III	Active, not recruiting	NCT04949256
ARRY-614+nivolumab vs ARRY-614+ nivolumab+ipilimumab	Solid Tumors	Phase Ib	Active, not recruiting	NCT04074967
ES002023 (Anti-CD39 Antibody)	Solid Tumors	Phase I	Completed	NCT05075564
AK119(Anti-CD73)+AK104(BsAbs)	Solid Tumors	Phase Ib/II	Completed	NCT04572152

To date, multiple guidelines for the management of irAEs have been issued, providing key suggestions for the handling of immune therapy-related toxicities (55, 56). The cornerstone of current irAEs management lies in early identification, comprehensive assessment, and prompt, appropriate intervention (53). Glucocorticoids, such as prednisolone or methylprednisolone, represent the first-line therapeutic agents for irAEs. Nevertheless, the diagnosis and treatment of severe and potentially life-threatening irAEs continue to pose significant clinical challenges. Owing to the absence of prospective clinical trial data regarding the use of immunosuppressive agents in the treatment of severe irAEs, current management approaches for these events are predominantly derived from aggregated analyses of limited-scale clinical studies and case reports. Martins et al. suggested that, according to the pathophysiological features of irAEs in patients, novel biological agents directed against key inflammatory mediators may be selectively employed for therapeutic intervention, such as monoclonal antibodies targeting TNF-α, IL-1, or IL-6 (57).

The decision to continue ICIs following the resolution of irAEs remains a subject of clinical discussion. According to the ASCO guidelines, management should be stratified based on the severity grading of adverse events (58): For Grade 1 irAEs, ICIs may be continued with close monitoring; for Grade 2 events, treatment should be withheld until symptoms resolve to Grade 1 or lower before considering reinitiation; Grade 3 irAEs necessitate treatment suspension; and in cases of severe or life-threatening Grade 4 irAEs, permanent discontinuation of ICIs is advised. Notably, any resumption of ICIs after treatment interruption due to irAEs should be made following a multidisciplinary specialist evaluation. During reinitiation, prior irAEs must be closely monitored for recurrence, and if recurrence occurs, permanent discontinuation should be strongly considered. Clinical decision-making should integrate patient-specific prognosis, tumor response, and the degree of irAE control to guide the implementation of individualized treatment strategies.

### 2.3 Advances in immunotherapy for the elderly and frail population

While immunotherapy has introduced promising therapeutic opportunities for numerous cancer patients, its application in elderly individuals (aged ≥ 65 years) presents clinical challenges. Elderly individuals, particularly those characterized by physical frailty, remain underrepresented in clinical research studies (59). Given the relatively favorable safety profile of ICIs, elderly patients who were previously deemed ineligible for anti-tumor therapy due to impaired performance status may now be reconsidered for ICIs treatment following a comprehensive reassessment. However, there remains a significant gap in dedicated research for elderly patients with ESCC. Currently, RAMONA stands as the only phase II study specifically designed for the geriatric population. Its results indicate that the nivolumab plus ipilimumab regimen offers significant clinical benefits with a reliable safety profile for elderly patients with refractory ESCC compared to chemotherapy (median OS: 7.2 vs. 5.9 months, P = 0.0063; grade ≥3 TRAE rate: 20%) (29). In subgroup analyses of elderly patients across multiple phase III trials investigating first- through later-line therapies for advanced ESCC, immunotherapy—whether administered as monotherapy or in combination with chemotherapy—consistently showed a trend toward improved OS versus control groups, as reflected by hazard ratios all below 1 (see **Tables 1**, **2** for detailed analyses of elderly subgroups). Therefore, for elderly patients without severe comorbidities, immunotherapy—particularly monotherapy or combinations with low-intensity chemotherapy—can be considered a reasonable treatment option. However, it must be noted that the conclusions from these phase III studies are primarily derived from subgroup analyses not stratified by age. The enrolled patients were generally younger, with elderly patients accounting for only 32.6%–48% of the study populations. Furthermore, dedicated safety data analyses specifically for this population are lacking. This limitation not only weakens the generalizability of the results to the elderly population but also makes it difficult to accurately assess the benefits and risks for this group in clinical practice. Consequently, caution is still warranted when applying existing evidence to elderly patients.

## 3 Biomarkers for predicting the efficacy of immune therapy

PD-L1 has been established as a biomarker for selecting patients with EC who may benefit from immunotherapy; however, its predictive value remains limited. It is generally accepted that patients with high PD-L1 expression derive greater benefit from ICIs (60), yet some studies have also found that patients with low PD-L1 expression can still benefit from such treatment (7, 36). Furthermore, inconsistencies across studies in the scoring systems, detection antibodies, and positivity thresholds pose challenges for data integration and interpretation. Currently, three PD-L1 expression scoring systems are used in ESCC: the TPS, which is determined by the percentage of viable tumor cells showing partial or complete membrane staining relative to all viable tumor cells; the CPS, calculated by dividing the number of PD-L1-stained cells (including tumor cells, lymphocytes, and macrophages) by the total number of viable tumor cells, multiplied by 100; and the TAP score, which assesses the percentage of tumor area occupied by PD-L1-stained tumor and immune cells (61). EGA and ESCC exhibit distinct patterns of PD-L1 expression. In ESCC, PD-L1 is commonly expressed on tumor cells, whereas in EGA, it is primarily expressed on cells within the tumor microenvironment (34, 62). This suggests that TPS may be a valid biomarker for ESCC, but not applicable to EGA. However, for the sake of operational simplicity, it is reasonable to uniformly apply CPS analysis for both EGA and ESCC, as the CPS scoring system can encompass TPS-positive tumors. Regarding detection methods for PD-L1, the testing platforms employed also vary across different studies. Adding to the complexity, the cutoff values defining PD-L1 positivity in clinical trials have not been standardized. For instance, studies related to pembrolizumab often use CPS ≥1 or ≥10 as cutoff values, whereas studies on nivolumab typically set a cutoff of 1%. Importantly, PD-L1 expression itself exhibits significant clinical heterogeneity and dynamic evolutionary characteristics. First, expression patterns differ among pathological subtypes. Second, PD-L1 expression is not static and can change dynamically during disease progression. Driven by therapeutic pressure (such as chemotherapy, radiotherapy, or targeted therapy) or tumor clonal evolution, patients who were initially PD-L1 negative may convert to positive, and vice versa. This spatiotemporal heterogeneity introduces uncertainty into the assessment results from a single biopsy and partly explains why some patients with low PD-L1 expression can still benefit from ICI therapy. Therefore, dynamically monitoring PD-L1 expression levels during immunotherapy holds promise for more accurately reflecting the true state of the tumor immune microenvironment and providing crucial information for subsequent treatment decisions (63). However, it remains unclear how to interpret discordant results (for example, whether to base treatment decisions on the lowest or highest CPS value).

In addition to PD-L1, patients with EC should also undergo testing for TMB and MSI. Similar to other cancer types, MSI-H and TMB-H are also robust biomarkers for predicting the efficacy of immunotherapy in EC. However, the incidence of MSI-H in esophageal cancer is less than 8%, and there is currently no consensus on the definition of TMB cutoffs (64–66). These issues not only highlight the complex biological characteristics of EC but also underscore the necessity of continuously optimizing existing biomarkers and identifying novel predictive markers.

Currently, biomarkers developed based on genomic or multi-omics technologies show promising potential for application, but their clinical translation is still in the preliminary exploratory stage. In the ALTER-003 study, whole exome sequencing (WES) was performed on baseline tumor samples from 41 patients with ESCC, demonstrating that the mutational status of TP53, FAT1, and NOTCH3 genes was correlated with clinical response, patients harboring TP53 or FAT1 mutations in the absence of NOTCH3 mutations (TP53+/FAT1+/NOTCH3-) exhibited substantial clinical benefit from combination therapy with anlotinib and a PD-L1 inhibitor, achieving an ORR of 65.6%, a median PFS of 17.91 months, and a 73% reduction in the risk of disease progression or death (47). Translational research based on the global RATIONAL-302 cohort suggested that NOTCH1 mutations may serve as a potential predictive biomarker for PD-1 inhibitor therapy in ESCC, with NOTCH1-mutant patients deriving greater benefit from tislelizumab (OS: 18.4 months vs. 5.3 months; HR: 0.35) (67). The JUPITER-06 study confirmed that patients with immunogenic features are more likely to benefit from immunotherapy. This study established an Esophageal Cancer Immuno-oncology Classification (EGIC) based on genomic profiling of pre-treatment tumor specimens from 514 patients with advanced ESCC. The results showed that patients in the EGIC1 subgroup (favorable immunogenic features without oncogenic alterations) and EGIC2 subgroup (favorable immunogenic features or no oncogenic alterations) had significantly improved survival with anti-PD-1 plus chemotherapy, whereas no significant benefit was observed in the EGIC3 subgroup (unfavorable immunogenic features with oncogenic alterations) (15). Is there a detection method that can answer two questions simultaneously with a single analysis: whether a patient is suitable for immunotherapy, and if not, which alternative therapy might be beneficial? Through multi-omics integrated analysis of tumor tissues from 155 ESCC patients, this study classified ESCC into four molecular subtypes: Cell Cycle Activation (CCA), NRF2 Pathway Activation (NRFA), Immunosuppressed (IS), and Immunomodulated (IM). Each subtype exhibits distinct molecular characteristics and therapeutic implications: The CCA subtype is characterized by CDKN2A deletions and CCND1 amplifications, suggesting potential benefit from CDK4/6 inhibitor therapy; the NRFA subtype features SOX2-mediated regulation of the NRF2 pathway, providing a potential rationale for NRF2-targeted therapy; while both IS and IM subtypes show high immune cell infiltration, the types of infiltrating cells differ—the IM subtype demonstrates better response to ICIs, whereas patients with the IS subtype may benefit from ERBB2 (HER2)-targeted therapy (68).

**Figure 1:**
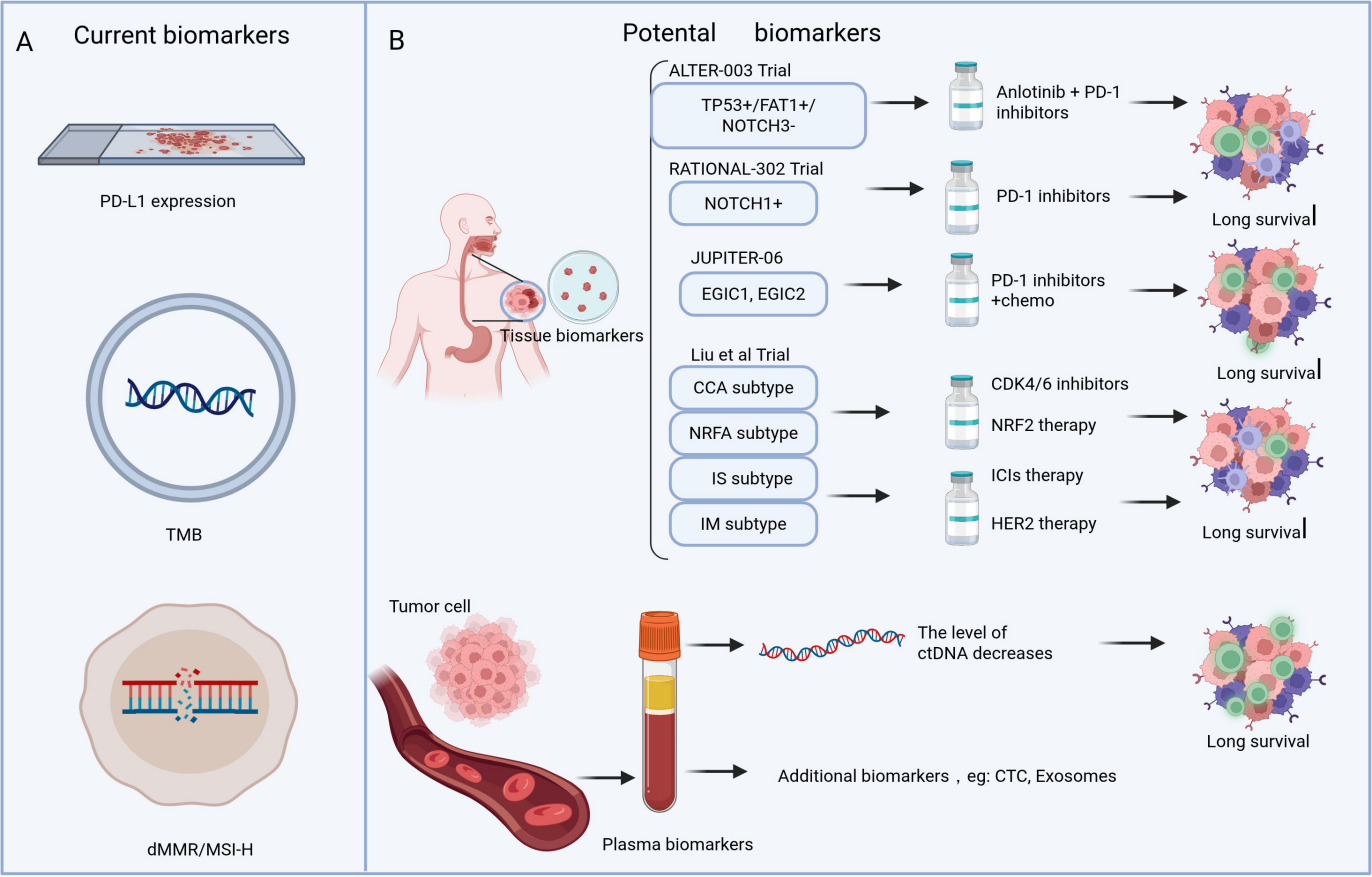
Panel **(A)** shows current biomarkers: PD-L1 expression, TMB, and dMMR/MSI-H. Panel **(B)** displays promising tissue and plasma biomarkers identified through clinical trials and confirms that patients receiving treatment guided by these biomarkers achieve longer survival outcomes.

Owing to the limited availability of tissue specimens, blood-based biomarker detection, commonly referred to as liquid biopsy, has emerged as a rapidly advancing approach in the field of tumor diagnosis and therapeutic management. Research confirms that circulating tumor DNA (ctDNA) detection, with its high sensitivity and specificity, can not only serve as a predictive biomarker to identify populations that may benefit from immunotherapy but also allow for dynamic monitoring of ctDNA changes to assess treatment response and prognosis (69, 70). Beyond ctDNA, other liquid biopsy approaches, including the detection of circulating tumor cells and exosomes, have also shown promising clinical potential in tumor prognosis assessment (**Figure 1**) (71, 72).

However, neither tissue-based biomarker systems nor liquid biopsy techniques have yet achieved true clinical translation. This situation is primarily constrained by the following factors: First, limited sample size. Existing studies generally have relatively small sample sizes, making it difficult to fully validate the universality and stability of biomarkers. Second, lack of standardization. Industry consensus has yet to be reached on the standardized testing procedures urgently needed for clinical translation, including critical aspects such as sample preprocessing and cut-off value determination. Third, interference from spatiotemporal heterogeneity. The inherent heterogeneity and dynamic evolutionary characteristics of tumors create the possibility of false-positive or false-negative results regardless of whether tissue or liquid biopsy is used. Fourth, economic burden constraints. The relatively high cost of biomarker testing, coupled with patients’ potential need for multiple tests during treatment, presents a practical challenge in clinical practice regarding how to alleviate patients’ financial burden.

To overcome these bottlenecks, future research should focus on the following directions: First, conduct high-quality clinical validation. Multi-center, large-scale prospective studies are needed to systematically evaluate the predictive efficacy and stability of novel biomarkers. Second, promote multi-technology integration and individualized strategies. Integrate liquid biopsy, genomics, proteomics and other technologies comprehensively, combined with patients’ genetic backgrounds and tumor biological characteristics, to construct individualized biomarker screening protocols while considering testing costs and clinical feasibility from the early stages of development. For testing items that have been rigorously validated and demonstrate clear benefits in health economics assessments, active efforts should be made to include them in health insurance coverage to reduce patients’ financial burden and enhance technology accessibility. Finally, establish standardized testing systems. Accelerate the development of industry standards covering the entire chain of sample processing, testing procedures, and result interpretation, providing an actionable implementation framework for the clinical translation of biomarkers. Only by bridging the “last mile” from technological development to clinical application can biomarker research be truly transformed into survival benefits for patients.

## 4 Resistance mechanisms in immunotherapy

Although immunotherapy has advanced significantly, drug resistance remains a major challenge. In 2020, the Society for Immunotherapy of Cancer categorized resistance to PD-1/PD-L1 inhibitors into two types (73): primary resistance, defined as disease progression occurring within 6 weeks to 6 months after the initiation of treatment, and secondary resistance, characterized by disease progression following an initial clinical response lasting at least 6 months. Primary resistance is primarily associated with mutations in oncogenic signaling pathways, impairments in neoantigen presentation, and aberrant epigenetic modifications. In contrast, acquired resistance is predominantly driven by the dynamic remodeling of the TME. Importantly, given the partial overlap between the mechanisms underlying primary and secondary resistance, these resistance mechanisms can be further categorized into tumor-intrinsic and tumor-extrinsic mechanisms.

Tumor neoantigens are tumor-specific antigens generated by gene mutations in tumor cells, capable of inducing a specific anti-tumor immune response in the body. TMB is used to quantify the number of non-synonymous mutations within the tumor genome and serves as an indirect indicator of neoantigen generation potential; generally, a higher mutational burden correlates with a greater probability of forming immunogenic neoantigens (74). Therefore, TMB is currently a commonly used surrogate marker for assessing tumor immunogenicity. The KEYNOTE-158 study demonstrated across various advanced solid tumors that patients with higher TMB and neoantigen burden are more sensitive to ICIs therapy compared to those with lower TMB (75). However, TMB cannot effectively predict treatment response to ICIs in all cancer types (76). Neoantigen deficiency resulting from genomic genetic alterations may be a significant mechanism mediating resistance to ICIs. Furthermore, studies in lung cancer and melanoma have identified mutations interfering with the antigen presentation pathway in tumor cells, including abnormalities in genes such as major histocompatibility complex (MHC) class I molecules, transporters associated with antigen processing, and beta-2-microglobulin. These have all been confirmed to be closely associated with impaired neoantigen presentation, defective T cell recognition of tumor antigens, and immunotherapy resistance (77–79).

Aberrant signaling and mutations in specific oncogenes represent critical intrinsic mechanisms underlying tumor immune resistance. Studies have confirmed that mutations in genes such as KRAS, EGFR, and mitogen-activated protein kinase (MAPK) not only drive tumor proliferation in colorectal cancer, lung cancer, and melanoma but also mediate resistance to PD-(L)1 inhibitors through various mechanisms, including suppressing IRF2 expression, recruiting myeloid-derived suppressor cells (MDSCs), downregulating PD-L1 expression, and reducing T cell infiltration (80–82). Furthermore, the loss of the tumor suppressor gene phosphatase and tensin homolog (PTEN) can remodel the TME by promoting the recruitment of immunosuppressive cells, thereby influencing the efficacy of immunotherapy. The Wnt/β-catenin signaling pathway is involved in regulating various fundamental biological processes in cells, and its aberrant activation is closely associated with tumor development, progression, invasion, metastasis, and immunotherapy resistance. In melanoma models, abnormal activation of Wnt/β-catenin signaling mediates resistance to PD-1/CTLA-4 inhibitors by suppressing T cell activation and chemokine secretion (83). Similarly, in melanoma, JAK1/2 gene mutations can impair T cell-mediated anti-tumor immune effects by interfering with IFN-γ signaling (84). Analogously, in ESCC, the long non-coding RNA (LncRNA) SNHG20 promotes ESCC cell progression by regulating the JAK-PD-L1 signaling axis (85). However, clinical studies on mutations in these key signaling pathways associated with ICIs resistance in ESCC remain scarce, and their specific mechanisms of action in ESCC require further elucidation and validation.

Epigenetic alterations in tumor cells also represent crucial endogenous mechanisms that contribute to the development of immune resistance. In ESCC cells, overexpression of the histone methyltransferase enhancer of zeste homolog 2 (EZH2) inhibits dendritic cell (DC) maturation through the promotion of vascular endothelial growth factor C (VEGFC) secretion and suppresses chemokine (C-X-C motif) ligand 9 (CXCL9) expression via catalysis of histone H3 lysine 27 trimethylation (H3K27me3), ultimately impairing CD8^+^ T cell infiltration into the TME (86). Additionally, DNA methyltransferases (DNMTs)-mediated demethylation modifications play a key role in the immune regulation of the TME. Studies have shown that DNMT1 is highly expressed in ESCC tumor tissues and cell lines. DNMT1 weakens the tumor’s ability to recruit T cells by inhibiting the expression of chemokines CXCL9 and CXCL10, thereby reducing the therapeutic efficacy of ICIs (87, 88).

In response to the above-mentioned endogenous drug resistance mechanisms, a number of novel drugs have entered the clinical research stage. For example, the combination treatment regimen utilizing the neoantigen-loaded dendritic cell vaccine NeoDC-Vac alongside ICIs is currently undergoing clinical evaluation in patients with advanced ESCC (NCT06675201). In the KRYSTAL-1 trial, the KRAS^G12C^ inhibitor adagrasib demonstrated an ORR of 35.1%, a mPFS of 7.4 months, and a favorable safety profile, with grade 3 or higher TRAEs occurring in less than 30% of patients (89). A phase Ib study assessing the p38-specific inhibitor ARRY-614, targeting the MAPK signaling pathway, in combination with ICIs for the treatment of refractory solid tumors is currently underway (NCT04074967). Furthermore, epigenetic modulatory agents, including DNA methyltransferase and histone deacetylase inhibitors, have demonstrated synergistic potential in enhancing the therapeutic efficacy of ICIs (88, 90).

In recent years, the TME has been recognized as a key mediator of resistance to ICIs through various immunosuppressive mechanisms. These mechanisms include the dysregulated expression of emerging immune checkpoint molecules, including TIM-3, LAG-3, and TIGIT(Please refer to section 2.1.3.), contributes to T cell exhaustion, the recruitment and activation of immunosuppressive cell populations, metabolic reprogramming, and the development of aberrant tumor vascular networks.

Within the TME, the roles of various immunosuppressive cells, such as regulatory T cells (Tregs), MDSCs, and tumor-associated macrophages (TAMs), in promoting tumor proliferation and inhibiting CD8^+^ T cell immune responses have been extensively elucidated, and their functions in ESCC have been further validated. For example, the interaction between progenitor-like CD8^+^ exhausted T cells and highly suppressive regulatory T cells (Hyper Tregs) within the TME can drive resistance to ICIs; Hyper Tregs may regulate CD8^+^ T cell activation via the CLEC2C-KLRB1 axis, forming an inhibitory immune cell interaction network centered on Hyper Tregs, thereby rendering the TME in a state of significant immunosuppression (91). Furthermore, in ESCC patients resistant to immunotherapy, the MIF-CXCR4 interaction has been observed to interfere with germinal center reactions by competitively antagonizing the CXCL12-CXCR4 signaling axis, thereby attenuating B cell-mediated anti-tumor immune responses and ultimately mediating immune resistance (92). However, effective clinical intervention strategies targeting these immunosuppressive cells and key molecules are currently lacking. Recent research in EC has revealed that GPR84 is specifically highly expressed on MDSCs. Targeting GPR84 holds promise for enhancing the response to anti-PD-1 immunotherapy in EC and potentially other malignancies, offering a potential strategy for reversing immune resistance (93).

Angiogenesis plays an essential role in the initiation, progression, and metastasis of malignant tumors. Within the TME, a variety of growth factors and their receptors are critically involved in angiogenesis, such as hypoxia-inducible factor (HIF), vascular endothelial growth factor (VEGF), VEGF receptors, fibroblast growth factor-2 (FGF2), transforming growth factor-α (TGF-α), and platelet-derived growth factor (PDGF). Among these factors, the VEGF family is universally acknowledged as a central regulator of angiogenesis. Among 124 patients with ESCC, 24%-74% exhibited overexpression of VEGF (94). The combined use of ICIs and therapies targeting VEGF/VEGF receptors is considered a synergistic treatment strategy that may positively modulate the TME (95). For detailed information regarding the relevant clinical trials, readers are referred to the sections on advancements in first-line and subsequent-line therapeutic strategies for advanced ESCC in Parts 2 and 3 of this manuscript.

Metabolic reprogramming is one of the hallmark features of tumors. It refers to the process by which tumor cells re-adjust and optimize their metabolic pathways during their occurrence and development to adapt to changes in the microenvironment and meet the demands of their rapid proliferation. In EC, it is mainly manifested as enhanced glycolysis and metabolic abnormalities. Tumor cells fulfill their elevated energy requirements through the upregulation of the glycolytic pathway, a metabolic phenomenon referred to as the “Warburg effect”. Research indicates that in ESCC, numerous glycolysis-associated enzymes exhibit substantial alterations. Among them, pyruvate kinase M2 (PKM2) serves as a key regulatory enzyme in the glycolytic pathway. Elevated expression levels of PKM2 have been observed in both tumor tissues and exosomes derived from the plasma of patients with ESCC. Overexpression of PKM2 can promote lactate accumulation, forming an immunosuppressive microenvironment, thereby inhibiting T cell function and weakening the body’s anti-tumor immune response (96, 97). CAR-T cell therapy targeting CD276 is capable of modulating this pathway and reversing immune resistance in ESCC (98). Hypoxia within the TME represents another critical component of tumor metabolic dysregulation. In studies on gastric cancer and EC, it was found that hypoxia exerts dual immunosuppressive effects via hypoxia-inducible factor-1α (HIF-1α)-dependent mechanisms: firstly, by activating the adenosine metabolic pathway (CD39/CD73/A2AR), which leads to T cell exhaustion; secondly, by promoting aberrant angiogenesis, thereby further enhancing immunosuppression within the TME (99). Building upon these findings, current research efforts are focused on investigating the synergistic combination of anti-PD-L1 therapy with anti-angiogenic therapy to promote vascular normalization (47). Furthermore, multiple phase I/II clinical trials targeting the adenosine pathway (such as CD39, CD73, A2AR) are underway (NCT05075564, NCT04572152).

**Figure 2:**
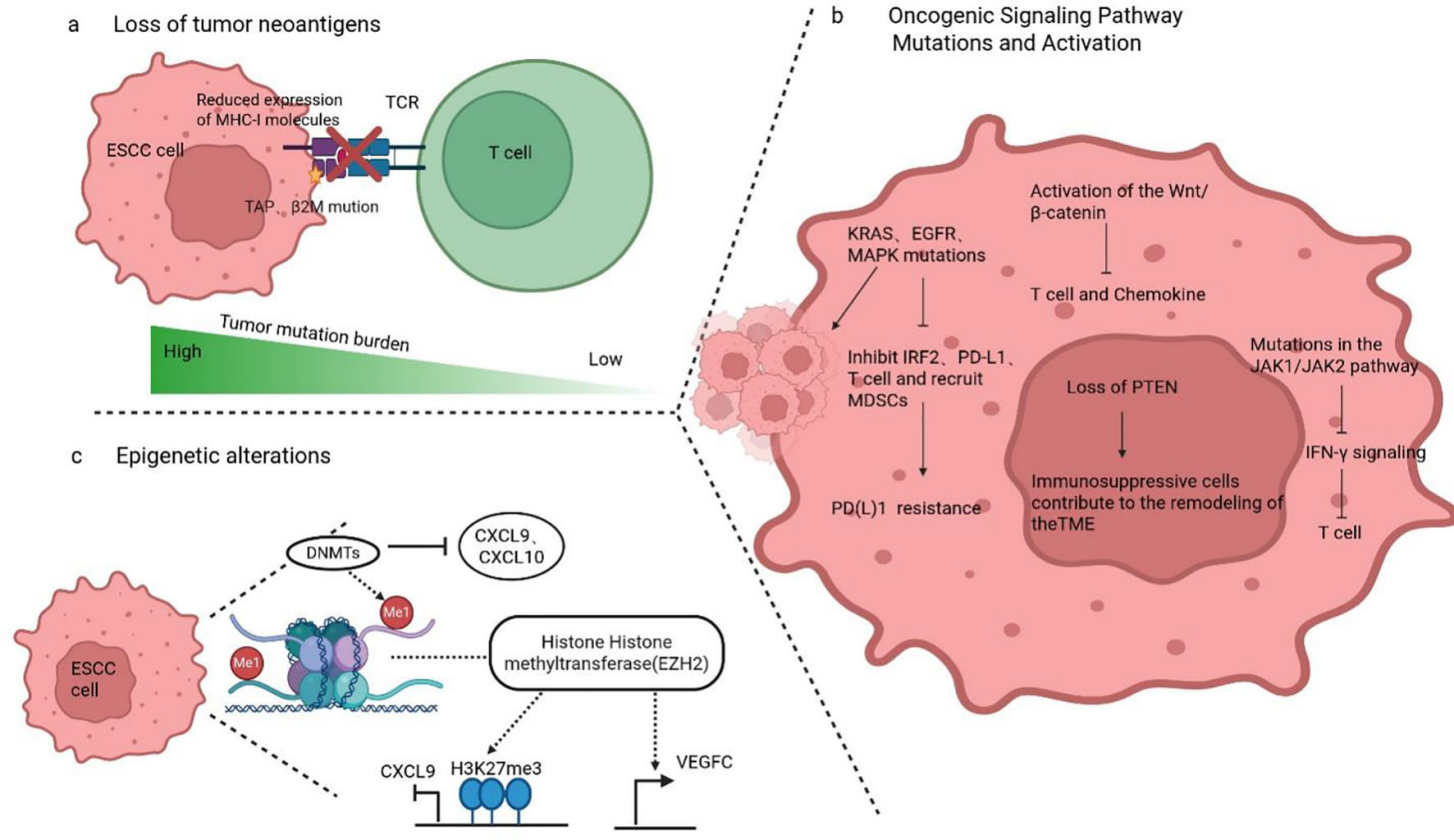
**(A)** Interference with MHC-I, TAP, and β2M expression leading to loss of tumor neoantigens; **(B)** Aberrant activation and mutation of oncogenic signaling pathways affecting cell signaling, PD-L1 resistance, and immunosuppression; **(C)** Epigenetic alterations (involving DNA methylation and histone modification) resulting in reduced chemokine production and increased VEGFC expression.

**Figure 3:**
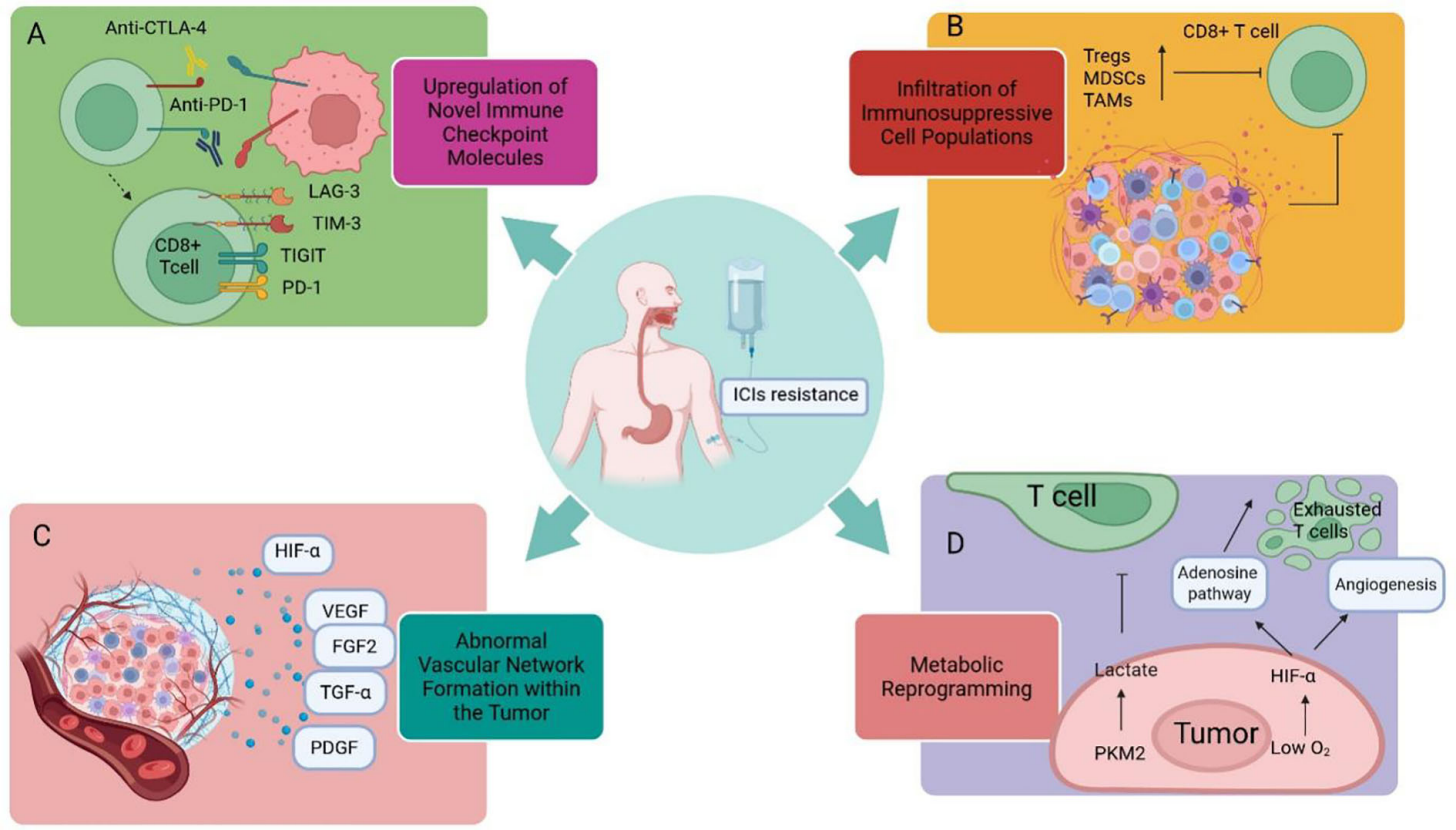
**(A)** Upregulation of novel immune checkpoint molecules; **(B)** Infiltration of immunosuppressive cell populations; **(C)** Formation of abnormal vascular networks within the tumor; **(D)** Metabolic reprogramming leading to T cell exhaustion.

In summary, immune resistance represents a complex network arising from the interplay between intrinsic tumor characteristics and the surrounding microenvironment, and it may manifest at any stage of therapy. Regardless of whether it is driven by a single dominant mechanism or the synergistic action of multiple mechanisms, immune resistance ultimately results in therapeutic failure. In-depth analysis of this mechanism will help achieve precise stratification of patients and optimization of treatment plans, providing guidance for the combination strategies of ICIs, as well as the development and design of new drugs and trials (**Figures 2**, **3**; **Tables 1**–**3**).

## 5 Conclusions and perspectives

The advent of immunotherapy has profoundly reshaped the treatment landscape for advanced ESCC. From second-line to first-line settings, and from monotherapy to combination regimens, immunotherapeutic strategies centered on PD-1/PD-L1 inhibitors have established their standard position, delivering durable survival benefits for patients. Concurrently, pioneering explorations into novel combination strategies (e.g., with anti-angiogenic agents, TIGIT inhibitors), BsAbs, and CAR-T cell therapies offer potential for further improving treatment outcomes.

Despite the progress made in immunotherapy for advanced ESCC, several core challenges in clinical practice demand urgent solutions. First, the predictive value of biomarkers remains limited. PD-L1 testing suffers from inconsistencies across scoring systems, detection platforms, and cut-off values. Biomarkers like TMB and MSI have constrained applicability in ESCC. Novel multi-omics biomarkers and liquid biopsy technologies are still in the exploratory clinical phase, hindered by small sample sizes, lack of assay standardization, tumor spatiotemporal heterogeneity, and high costs, impeding their integration into clinical decision-making frameworks. Second, the mechanisms of immune resistance are complex and not yet fully elucidated. Intrinsic tumor factors such as neoantigen deficiency, aberrant signaling pathways, and epigenetic alterations, along with extrinsic factors mediated by the TME including immunosuppression, metabolic reprogramming, and abnormal angiogenesis, collectively contribute to primary or acquired resistance, posing a key barrier to enhancing immunotherapy efficacy. Third, the therapeutic needs of special populations remain inadequately addressed. Elderly and frail patients are underrepresented in clinical studies, leading to a lack of dedicated treatment regimens and safety data, making it difficult to accurately assess their benefit-risk profile in clinical application. Fourth, obstacles persist in the clinical translation of novel immunotherapies. Modalities like BsAbs and CAR-T therapies have yet to demonstrate significant “enhanced efficacy with reduced toxicity” advantages in ESCC, and some combination regimens lack confirmatory Phase III data to support their efficacy.

Future advancements in immunotherapy for advanced ESCC will pivot around the core directions of precision, personalization, and diversification, focusing on overcoming the following research and clinical application bottlenecks. First, intensify the development and clinical translation of biomarkers. Conduct multicenter, large-scale prospective studies to validate the predictive power of novel multi-omics biomarkers and liquid biopsy techniques. Accelerate the establishment of unified standardization systems covering the entire chain of sample processing, testing procedures, and result interpretation, while considering assay feasibility and cost-effectiveness. Promote the integration of rigorously validated biomarkers into routine clinical testing and health insurance coverage to enable precise patient stratification and individualized treatment planning. Second, deeply dissect the molecular mechanisms of immune resistance. Utilize multi-omics technologies to identify key resistance-related targets and signaling pathways. Explore combination strategies integrating immunotherapy with targeted therapy, epigenetic modulation, or metabolic intervention to reverse the immunosuppressive TME, thereby providing new therapeutic targets and regimens to overcome primary and acquired resistance. Third, focus on clinical research and treatment optimization for special populations. Design and conduct clinical trials specifically for elderly, frail, and patients with comorbidities to evaluate the efficacy and safety of different immunotherapeutic approaches, developing individualized, low-toxicity, and highly effective treatment strategies to enhance therapeutic accessibility and benefit for these groups. Fourth, accelerate the development and clinical validation of novel immunotherapies. Optimize the design and manufacturing processes of BsAbs and CAR-T therapies to address challenges like antigen heterogeneity in solid tumors and insufficient cell infiltration. Conduct head-to-head clinical trials to verify the superiority of novel combination regimens over standard immunochemotherapy, identifying strategies with genuine clinical value. Fifth, refine the comprehensive management system for irAEs. Utilize biomarkers for early prediction and stratified intervention of irAEs. Explore the application of novel biologic agents for managing severe irAEs, balancing the efficacy and safety of immunotherapy to further enhance patient treatment tolerability and quality of life.

Furthermore, the deep integration of multidisciplinary collaboration and translational medicine research will provide crucial support for advancing immunotherapy in advanced ESCC. By closely linking basic research with clinical practice, the transformation of laboratory discoveries into clinically accessible treatment modalities and detection technologies can be accelerated. Simultaneously, leveraging real-world evidence to complement the limitations of clinical trials will furnish a more comprehensive evidence base for the clinical application of immunotherapy.

In summary, immunotherapy has brought revolutionary survival benefits to patients with advanced ESCC and has become a core component of comprehensive treatment for this disease. With continuous advancements in biomarker development, gradual elucidation of resistance mechanisms, ongoing exploration of novel therapeutic strategies, and progressive refinement of clinical management systems, immunotherapy for advanced ESCC will steadily evolve towards greater precision, efficacy, and safety. This holds the promise of further improving patient response rates and long-term survival, ultimately achieving a paradigm shift from “population-based treatment” to “individualized precision therapy,” and offering increased hope for a cure to patients with advanced ESCC.
